# Effect of extended defects on photoluminescence of gallium oxide and aluminum gallium oxide epitaxial films

**DOI:** 10.1038/s41598-022-07242-z

**Published:** 2022-02-25

**Authors:** Jacqueline Cooke, Praneeth Ranga, Jani Jesenovec, John S. McCloy, Sriram Krishnamoorthy, Michael A. Scarpulla, Berardi Sensale-Rodriguez

**Affiliations:** 1 Department of Electrical and Computer Engineering, The University of Utah, Salt Lake City, UT 84112 USA; 2 Department of Materials Science and Engineering, The University of Utah, Salt Lake City, UT 84112 USA; 3 Materials Department, University of California, Santa Barbara, Santa Barbara, CA 93106-5050 USA; 4 Institute of Materials Research, Washington State University, Pullman, WA 99164-2920 USA; 5 Materials Science and Engineering Program, Washington State University, Pullman, WA 99164 USA

**Keywords:** Optical spectroscopy, Materials for devices

## Abstract

In this work, a systematic photoluminescence (PL) study on three series of gallium oxide/aluminum gallium oxide films and bulk single crystals is performed including comparing doping, epitaxial substrates, and aluminum concentration. It is observed that blue/green emission intensity strongly correlates with extended structural defects rather than the point defects frequently assumed. Bulk crystals or Si-doped films homoepitaxially grown on (010) β-Ga_2_O_3_ yield an intense dominant UV emission, while samples with extended structural defects, such as gallium oxide films grown on either (-201) β-Ga_2_O_3_ or sapphire, as well as thick aluminum gallium oxide films grown on either (010) β-Ga_2_O_3_ or sapphire, all show a very broad PL spectrum with intense dominant blue/green emission. PL differences between samples and the possible causes of these differences are analyzed. This work expands previous reports that have so far attributed blue and green emissions to point defects and shows that in the case of thin films, extended defects might have a prominent role in emission properties.

## Introduction

Gallium oxide is an ultra-wide bandgap semiconductor, with its most thermally and chemically stable phase being the monoclinic structure, β-Ga_2_O_3_. It has an indirect, fundamental bandgap near 276 nm (4.5 eV) though optical transition energies range from 282 to 253 nm (4.5 to 4.9 eV) depending on the crystallographic orientation due to the anisotropy of this material^1^. β-Ga_2_O_3_ has a large Baliga’s figure of merit which has increasingly garnered interest in various electronics and optoelectronics applications. Understanding and characterizing the material properties, including its defects, has been a priority, and photoluminescence (PL) has been under intense scrutiny in an attempt to define the mechanisms that generate the emissions in this material.

In general, PL spectra can be used to characterize the defects leading to radiative recombination processes within a specific material. In this regard, the PL spectra for β-Ga_2_O_3_ has generally been deconvoluted in three emission peaks: UV, blue, and green. However, it is notable that peak shapes from any point defect are expected to require more than a single Gaussian for a complex crystal structure like β-Ga_2_O_3_, possibly having an asymmetric shape and requiring a more complex model such as Huang-Rhys or Franck–Condon, which takes into consideration the vibrational broadening of the PL spectrum caused by electron–phonon coupling^2–4^. The sum of so many phonon replicas makes it challenging to fit the spectra uniquely. Adding energy level broadening from disorder or extended defects makes the deconvolution of the β-Ga_2_O_3_ PL spectra even more difficult. As such, in β-Ga_2_O_3_ with strong electron phonon coupling, there is little chance of being able to spectroscopically discern different types of defects from luminescence as any defect considered can result in a very wide luminescence band, even near 0 Kelvin. This has resulted in an intense debate in defining the defects and phenomenological explanations of electronic processes that cause these particular emissions^5–24^. Provided in the Supplementary Information is a comprehensive review of the literature.

Point defects have been the only explored and discussed potential source for the visible PL emission peaks in β-Ga_2_O_3_. Generally, there has been recent agreement on an intrinsic origin of UV luminescence and an extrinsic origin of visible emission. But, as far as can be determined, no previous works in the literature have discussed whether extended defects affect PL. Here, by systematically analyzing a series of thin-film samples, it is observed that extended defects substantially affect blue/green emissions and that this is correlated with poor structural quality samples. This analysis expands on previous reports that have so far attributed blue and green emissions to point defects and shows that in the case of thin films, extended defects may have a prominent role in emission properties.

## Experiment

### Sample preparation

All film samples were grown using metal–organic vapor-phase epitaxy (MOVPE) in an Agnitron Agilis reactor. All samples were grown using triethylgallium (TEGa) and O_2_ as the gallium and oxygen precursors, respectively, and silane as the Si dopant source when applicable. Growth details are listed in Table 1. Parameters used for all samples include a total molar flow of 15.53 μmol/min, argon flow rate of 1100 sccm, an oxygen flow rate of 500 sccm, and a chamber pressure of 15 Torr with a growth rate of around 6 nm/min. The substrates used for growth were either Fe-doped (-201) or (010) oriented β-Ga_2_O_3_ grown by Novel Crystal Technology. Otherwise, C-plane sapphire was used for growth and purchased from Cryscore. An unintentionally doped (UID) single crystal (-201) oriented β-Ga_2_O_3_ bulk sample grown using edge-defined film fed (EFG) technology was purchased from Novel Crystal Technology. Lastly, a single crystal (100) oriented 10% bulk aluminum-gallium oxide (AGO) sample, β-Al_0.2_Ga_1.8_O_3_ was grown using the Czochralski method. Details on the bulk AGO sample and growth can be found in^25^.

**Table 1:** Growth parameters for all the analyzed samples.

	Samples	Doping (cm^−3^)	Dopant	Orientation	Substrate	Film or crystal thickness (μm)	Growth temp. (°C)	TEGa flow rate (sccm)	TMAl molar ratio (push/purge/double dilution)
**Si series**									
	UID β-Ga_2_O_3_	3 × 10^16^	Si (UID)	(010)	Fe-doped β-Ga_2_O_3_	1	600	65	
	β-doped Ga_2_O_3_	1.5 × 10^17^	Si	(010)	Fe-doped β-Ga_2_O_3_	1	600	65	
	Heavy doped β-Ga_2_O_3_	5 × 10^18^	Si	(010)	Fe-doped β-Ga_2_O_3_	1	600	65	
**(-201) oriented series**									
	Bulk β-Ga_2_O_3_	1.9 × 10^17^	UID	(-201)	Bulk	670	Purchased from Novel Crystal Technology (EFG method)		
	β-Ga_2_O_3_ on β-Ga_2_O_3_	~ 10^16^	UID	(-201)	Fe-doped β-Ga_2_O_3_	0.350–0.400	650	65	
	β-Ga_2_O_3_ on sapphire	~ 10^16^	UID	(-201)	Sapphire	~ 0.400	810	65	

	Samples	% Concentration	Dopant	Orientation	Substrate	Film or crystal thickness (μm)	Growth temp. (°C)	TEGa flow rate (sccm)	TMAl molar ratio (push/purge/double dilution)
**AGO series**									
	β-Ga_2_O_3_ on β-Ga_2_O_3_		UID	(010)	Fe-doped β-Ga_2_O_3_	0.350–0.400	650	65	
	10% AGO on β-Ga_2_O_3_	10 (PMR)	Al	(010)	Fe-doped β-Ga_2_O_3_	0.350–0.400	650	58.5	10/200/84
	25% AGO on β-Ga_2_O_3_	25 (EDS)	Al	(010)	Fe-doped β-Ga_2_O_3_	0.350–0.400	650	52	15/100/1.5
	10% bulk AGO	10 (XRF)	Al	(100)	Bulk	~ 2000	From Washington State University (Czochralski method)		
	β-Ga_2_O_3_ on sapphire		UID	(-201)	Sapphire	~ 0.400	810	65	
	2% AGO on Sapphire	2 (XRD)	Al	(-201)	Sapphire	~ 0.400	810	61.6	10/200/42
	10% AGO on Sapphire	10 (XRD)	Al	(-201)	Sapphire	~ 0.400	810	58.5	10/150/64
	28% AGO on Sapphire	28 (XRD)	Al	(-201)	Sapphire	~ 0.400	810	48.7	30/100/43.5

Three series of samples were analyzed. The first series includes three film samples with varying Si-doping (used to make β-Ga_2_O_3_ more conductive and due to its strong PL signature): UID (~ 10^16^ cm^−3^), ~ 10^17^ cm^−3^, and ~ 10^18^ cm^−3^, with required silane flows determined using silicon secondary ion mass spectrometry (SIMS) calibrations^26^. All samples in the series are (010) oriented Si-doped β-Ga_2_O_3_ film grown on Fe-doped β-Ga_2_O_3_. The second series also contains three samples and compares (-201) β-Ga_2_O_3_. This series includes a bulk crystal of (-201) UID β-Ga_2_O_3_, a (-201) UID β-Ga_2_O_3_ film grown on Fe-doped β-Ga_2_O_3_, and a (-201) UID β-Ga_2_O_3_ film grown on sapphire. The last series compares (010) oriented (Al_x_Ga_1−x_)_2_O_3_ films, i.e., AGO of varying Al concentrations, grown on Fe-doped β-Ga_2_O_3_. These are compared to a (100) oriented bulk 10% AGO (β-Al_0.2_Ga_1.8_O_3_) crystal. The second half of the series compares (-201) oriented 0%, 2%, 10%, and 28% AGO films grown on sapphire.

X-ray diffraction (XRD) was performed on all epitaxial AGO films. For AGO films grown on sapphire, the value obtained from XRD is used to define the aluminum concentration. Note that the AGO samples grown on Fe-doped β-Ga_2_O_3_ yielded Al composition values that were much larger than expected: 20% for the nominally 10% AGO sample (i.e., grown using a molar ratio that should have yielded ~ 10% Al) and 30% for the nominally 25% AGO sample (i.e., grown using a molar ratio that should have produced ~ 20% Al). This is likely due to strain and relaxation that occurs in the homoepitaxial film, which was found riddled with extended defects using STEM (shown in the results section). Furthermore, EDS measurements verified an aluminum concentration of 25% for the nominal 25% AGO sample. Therefore, for the 10% AGO sample, an Al composition value from the precursor molar ratio (PMR) during growth is used instead of the value obtained from XRD. The bulk AGO sample was measured by Washington State University using X-ray fluorescence (XRF) and found to have an Al composition of 10%^25^.

### Characterization

Photoluminescence was performed using ultrafast (fs) pulses from a wavelength-tunable (690–1040 nm or 1.8–1.2 eV) Ti:Sapphire (Coherent Chameleon Vision Ultra) laser, which passed through a third-harmonic generator (Coherent Harmonics). The laser was then polarized using a linear polarizer (Glan-Laser alpha-BBO polarizer prism, 210–450 nm or 5.9–2.76 eV) followed by a zero-order half-wave plate to control the polarization angle of the laser. The laser excited the sample at normal incidence within an integration sphere. The PL spectra were collected at room temperature using an optical fiber connected to a broadband spectrometer in the range of 300–800 nm or 4.13–1.55 eV (Avasoft AvaSpec dual-channel spectrometer).

Samples were measured using an excitation of 235 nm (5.27 eV) (236 nm or 5.25 eV for AGO grown on sapphire), 254 nm (4.88 eV), and 267 nm (4.64 eV). Polarization was altered from 0 to 180 degrees with a 15-degree step size. The collected data was corrected to remove the response caused by the spectrometer and eliminate the grating and detector response, so as to extract the response of the sample itself. The data collected was corrected for the spectrometer spectra using the Ocean Insight HL-3 plus visible-near infrared (VIS–NIR) light source. The spectrum for this light source was calibrated by Shanghai Calibration Laboratory. The calibrated blackbody radiant energy spectra from the laboratory was divided by the spectra collected by our spectrometer to get a correction factor. This correction factor was then applied to the spectra collected for all samples except AGO samples grown on sapphire. The AGO samples grown on sapphire used a quartz-tungsten-halogen (QTH) lamp (Oriel Instruments), whose spectrum is well defined as a blackbody source given by Plank’s radiation law. The measured light source was fitted to this radiation law, and a temperature correction factor was applied for the corrected data. These correction factors were then applied to measured PL data for the AGO samples grown on sapphire. All the corrected spectra were then normalized by the power measured (using a Newport optical power meter 1830-C) and integration time (which was kept at 5 s for all samples).

## Results

### Si series

**Figure 1:**
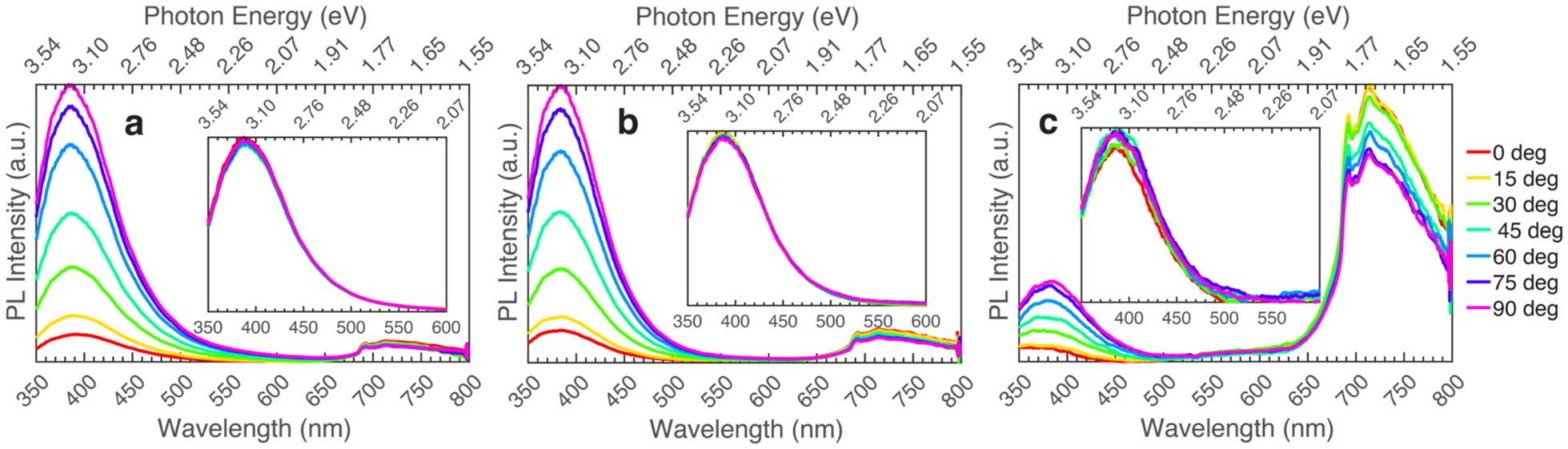
Polarized photoluminescence of β-Ga_2_O_3_ grown on (010) Fe-doped β-Ga_2_O_3_ excited at 267 nm (4.64 eV). Insets show data for the same samples but excited at 235 nm (5.27 eV). (**a**) UID (~ 10^16^ cm^−3^) β-Ga_2_O_3_ grown on (010) Fe-doped β-Ga_2_O_3_, (**b**) Si-doped (1.5 × 10^17^ cm^−3^) β-Ga_2_O_3_ grown on (010) Fe-doped β-Ga_2_O_3_, (**c**) Si-doped (5 × 10^18^ cm^−3^) β-Ga_2_O_3_ grown on (010) Fe-doped β-Ga_2_O_3_.

Depicted in Fig. 1 is the measured polarized PL for all the samples in the series excited above and below the bandgap. Panels (a-c) correspond to the polarized emission spectrum of each sample: UID (~ 10^16^ cm^−3^) β-Ga_2_O_3_ grown on Fe-doped (010) oriented β-Ga_2_O_3_ substrate, Si-doped (1.5 × 10^17^ cm^−3^) β-Ga_2_O_3_ grown on Fe-doped (010) oriented β-Ga_2_O_3_ substrate, and Si-doped (5 × 10^18^ cm^−3^) β-Ga_2_O_3_ grown on Fe-doped (010) oriented β-Ga_2_O_3_ substrate. For the main plots in each panel, an excitation wavelength of 267 nm (4.64 eV) is employed, whereas the insets correspond to 235 nm (5.27 eV) excitation wavelength. All three samples in the Si series show a dominant UV emission, with peak emission around 385 nm (3.22 eV) for excitation both below (267 nm or 4.64 eV) and above (235 nm or 5.27 eV) bandgap. Polarization dependence of emission is seen below the bandgap for all three samples. The observed red emission starting around 700 nm (1.77 eV) is due to the Fe-doped β-Ga_2_O_3_ substrate on which the Si-doped β-Ga_2_O_3_ films were grown^27,28^.

**Figure 2:**
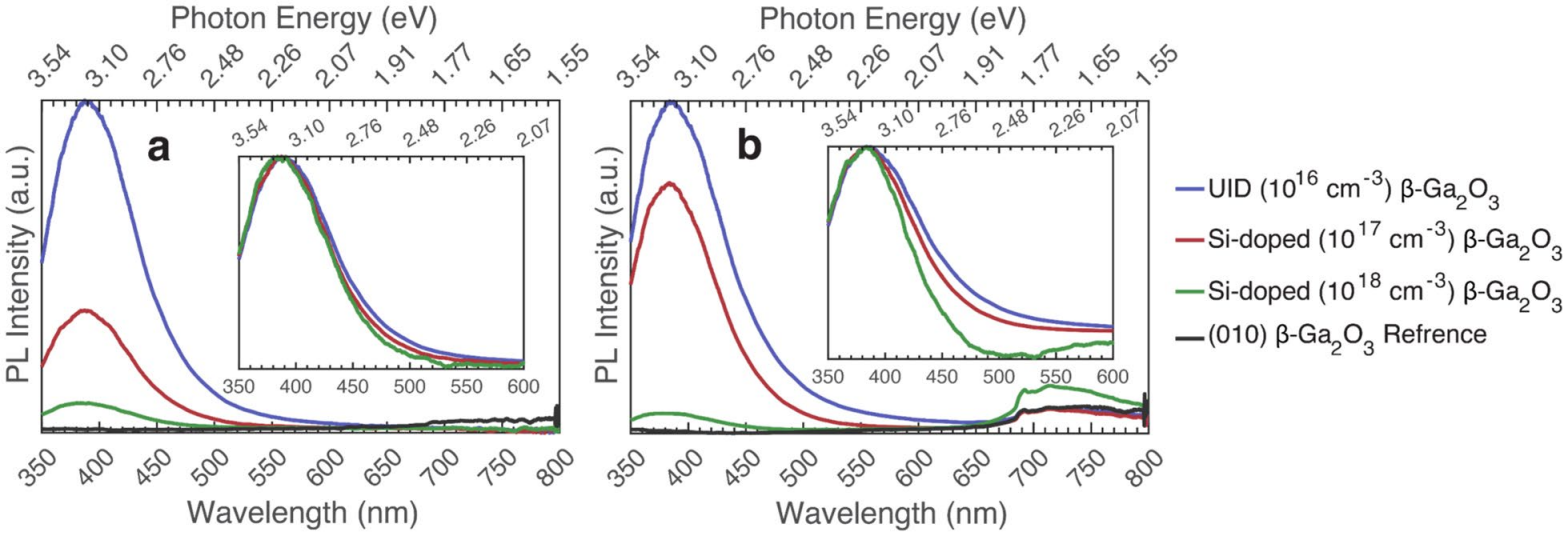
Photoluminescence comparison (taken at max polarization) of Si-doped β-Ga_2_O_3_ series grown on (010) Fe-doped β-Ga_2_O_3._ Insets show the same data but normalized to peak intensity. (**a**) Excited at 235 nm (5.27 eV), (**b**) Excited at 267 nm (4.64 eV).

The PL of the varying Si-doped samples grown on Fe-doped β-Ga_2_O_3_ is compared in Fig. 2, excited above and below the bandgap. This series of samples revealed that increasing Si doping leads to a decrease in overall PL intensity. This is likely due to an increase in non-radiative recombination^7,13,29^. The dominant emission within the UV region (385 nm or 3.22 eV) does not change between the samples. This is possibly due to the samples having a homogeneous film with limited extended defects. Transmission electron microscopy (TEM) images in previous reports show that β-Ga_2_O_3_ films grown on (010) β-Ga_2_O_3_ continue to have the same crystal structure as the substrate, with no stacking faults or other extended defects^30,31^. However, there is a decrease in the blue luminescence (from 400 to 500 nm or 3.1 to 2.48 eV) as the Si-doping in the samples increases. Other reports have observed and explained this observation as a decrease in donor–acceptor pairs, where the oxygen vacancy (V_O_) donors are reduced^7,13,29,32^. The same phenomenon has been seen in other reports for Sn-doped β-Ga_2_O_3,_ which measured PL before and after annealing, and showed a dominant UV emission around 380 nm (3.26 eV) for both cases^33–35^. Quenching of the UV emission, relative to the blue/green after annealing, was seen resulting from an increase in non-radiative recombination (conductivity), which was suggested to be due to a change in the formation of oxygen vacancies and gallium vacancy-oxygen vacancy complexes^33,35^.

### (-201) Orientation series

**Figure 3:**
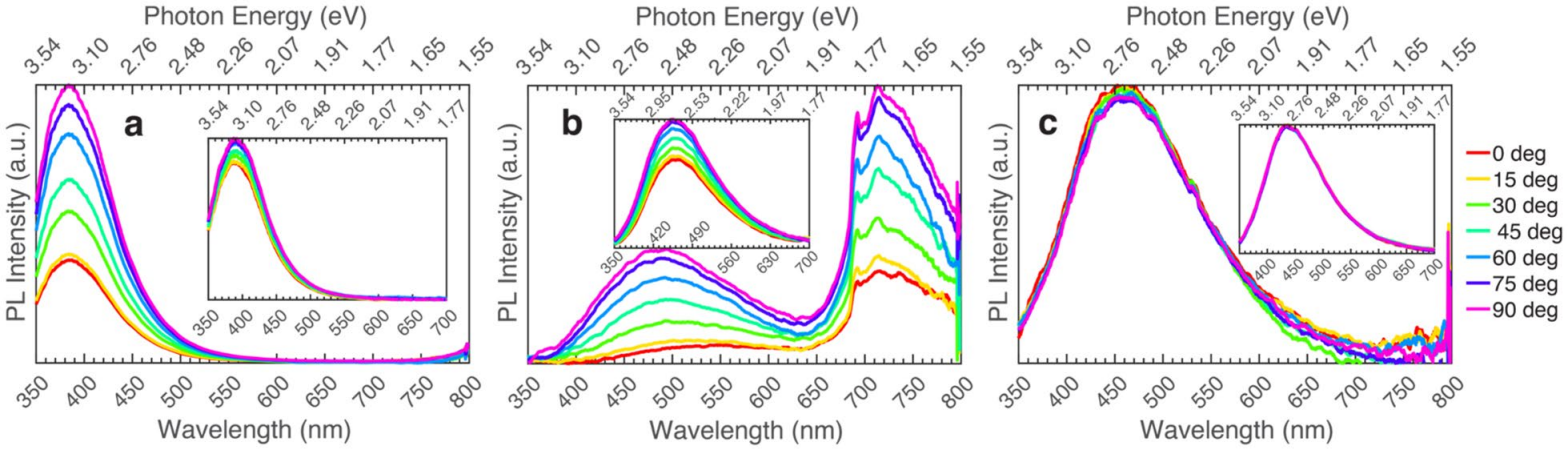
Polarized photoluminescence of β-Ga_2_O_3_ grown on different substrates excited at 267 nm (4.64 eV). Insets show data for the same samples but excited at 235 nm (5.27 eV). (**a**) Bulk (-201) β-Ga_2_O_3_, (**b**) β-Ga_2_O_3_ grown on (-201) Fe-doped β-Ga_2_O_3_, (**c**) (-201) β-Ga_2_O_3_ grown on c-plane sapphire.

The three (-201) oriented samples in this series show several differences even though all the samples are UID β-Ga_2_O_3_. Depicted in Fig. 3 is the polarized PL of (-201) UID β-Ga_2_O_3_ film grown on Fe-doped β-Ga_2_O_3,_ a (-201) UID β-Ga_2_O_3_ bulk crystal, and a (-201) UID β-Ga_2_O_3_ film grown on sapphire, all excited above and below bandgap. The bulk crystal and the β-Ga_2_O_3_ film grown on β-Ga_2_O_3_ both show polarization dependence of emission with weak dependence above (235 nm or 5.27 eV) and intense dependence below (267 nm or 3.22 eV) bandgap. But the β-Ga_2_O_3_ film grown on the sapphire substrate shows no polarization no matter what excitation is used, as seen in Fig. 3. This is attributed to rotational domains generated in the growth process for β-Ga_2_O_3_ films when grown on sapphire, which is the development of a twofold crystal on a threefold substrate as described by Ghadbeigi et al*.* as well as in several other studies^36–38^. As such, polarization dependence is not expected, and the PL at each polarization should be an average arising from the rotationally misaligned crystalline domains.

**Figure 4:**
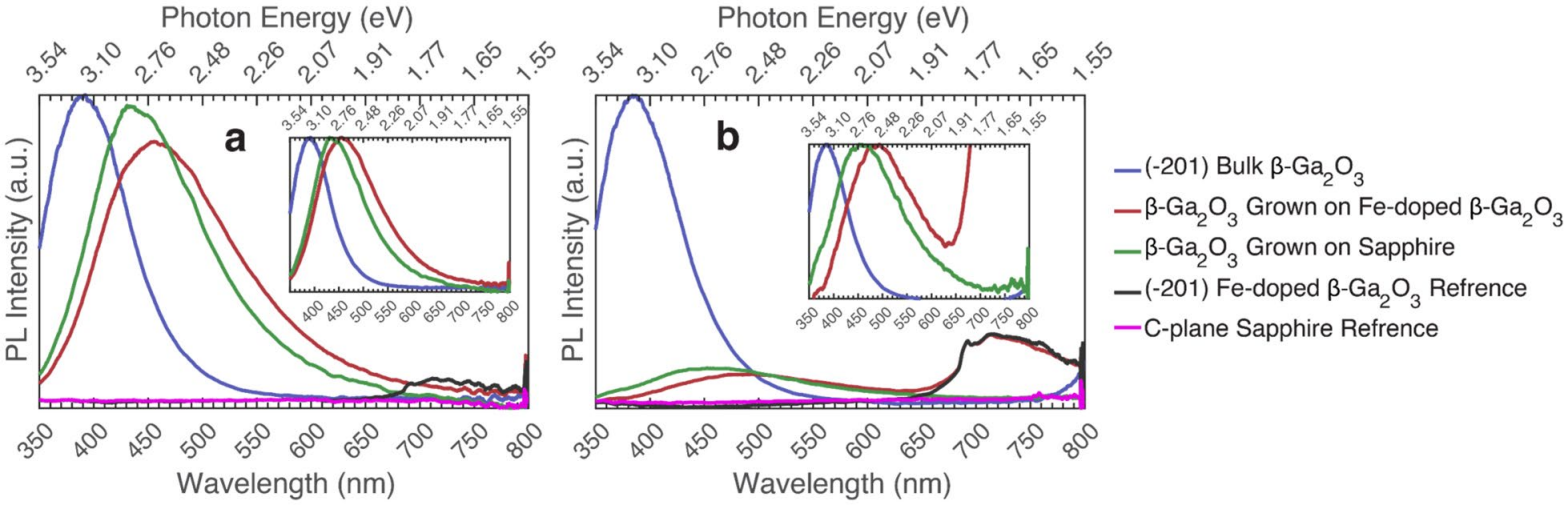
Photoluminescence comparison (taken at max polarization) of β-Ga_2_O_3_ grown on different substrates. Insets show the same data but normalized to the peak values. (**a**) Excited at 235 nm (5.27 eV), (**b**) Excited at 267 nm (4.64 eV).

Depicted in Fig. 4 is a PL comparison of all the (-201) UID β-Ga_2_O_3_ samples excited above and below the bandgap. As seen, the PL intensity from all samples is comparable above (235 nm or 5.27 eV) bandgap; but, below (267 nm or 4.64 eV) bandgap, the intensity of the epitaxial films diminishes considerably compared to the bulk sample. Furthermore, the bulk sample shows a dominant UV peak around 390 nm (3.18 eV) both above and below the bandgap. However, the β-Ga_2_O_3_ films grown on β-Ga_2_O_3_ and on sapphire show a dominant blue emission around 460 nm (2.7 eV) and 430 nm (2.88 eV) above and below bandgap, respectively. Similar observations were made in other reports for β-Ga_2_O_3_ grown on sapphire showing a dominant emission around 440 nm (2.82 eV)^30,39,40^. The difference in dominant emission between samples of this series is likely due to extended defects ruling the emission of the PL found in epitaxial films. The studied bulk β-Ga_2_O_3_ crystals are assumed to have a small density of extended defects compared to an epitaxially grown film due to being a single crystal. Furthermore, note that the PL emission for the bulk β-Ga_2_O_3_ and the Si-doped series both have a dominant UV peak, even though the Si-doped series was epitaxially grown and had varying amounts of point defects in the samples, which did not cause a shift in the dominant UV peak. As previously shown, β-Ga_2_O_3_ grown on (010) β-Ga_2_O_3_ produces a homogeneous film with no stacking faults or extended defects^30,31^.

On the other hand, both the epitaxial films in the (-201) oriented series are riddled with extended defects, as has been documented in several reports. Along with multiple reports, including Ghadbeigi et al*.* which showed rotational domains^36–39^, TEM images of (-201) β-Ga_2_O_3_ grown on sapphire revealed multiple extended defects in these types of samples^31,37–39,41–44^. Furthermore, Eisner et al*.* studied a similarly MOVPE grown sample like the β-Ga_2_O_3_ film grown on (-201) Fe-doped β-Ga_2_O_3_. Using TEM, they showed that the film quality was poor and exhibited multiple extended defects throughout the sample^45^. Additionally, the Si-doped series has comparable growth conditions to the (-201) β-Ga_2_O_3_ film grown on a differently oriented β-Ga_2_O_3_ substrate, suggesting that growth conditions that could change the number of a specific type of point defect in the sample could be ruled out. The only difference between the Si-doped series and (-201) grown on Ga_2_O_3_ is that β-Ga_2_O_3_ grown on (010) β-Ga_2_O_3_ yields a homogeneous film while β-Ga_2_O_3_ grown on (-201) β-Ga_2_O_3_ yields a poor-quality film riddled with extended defects. Thus, we hypothesize a strong correlation between the dominant blue peak seen in (-201) β-Ga_2_O_3_ film samples and extended defects.

### AGO series

**Figure 5:**
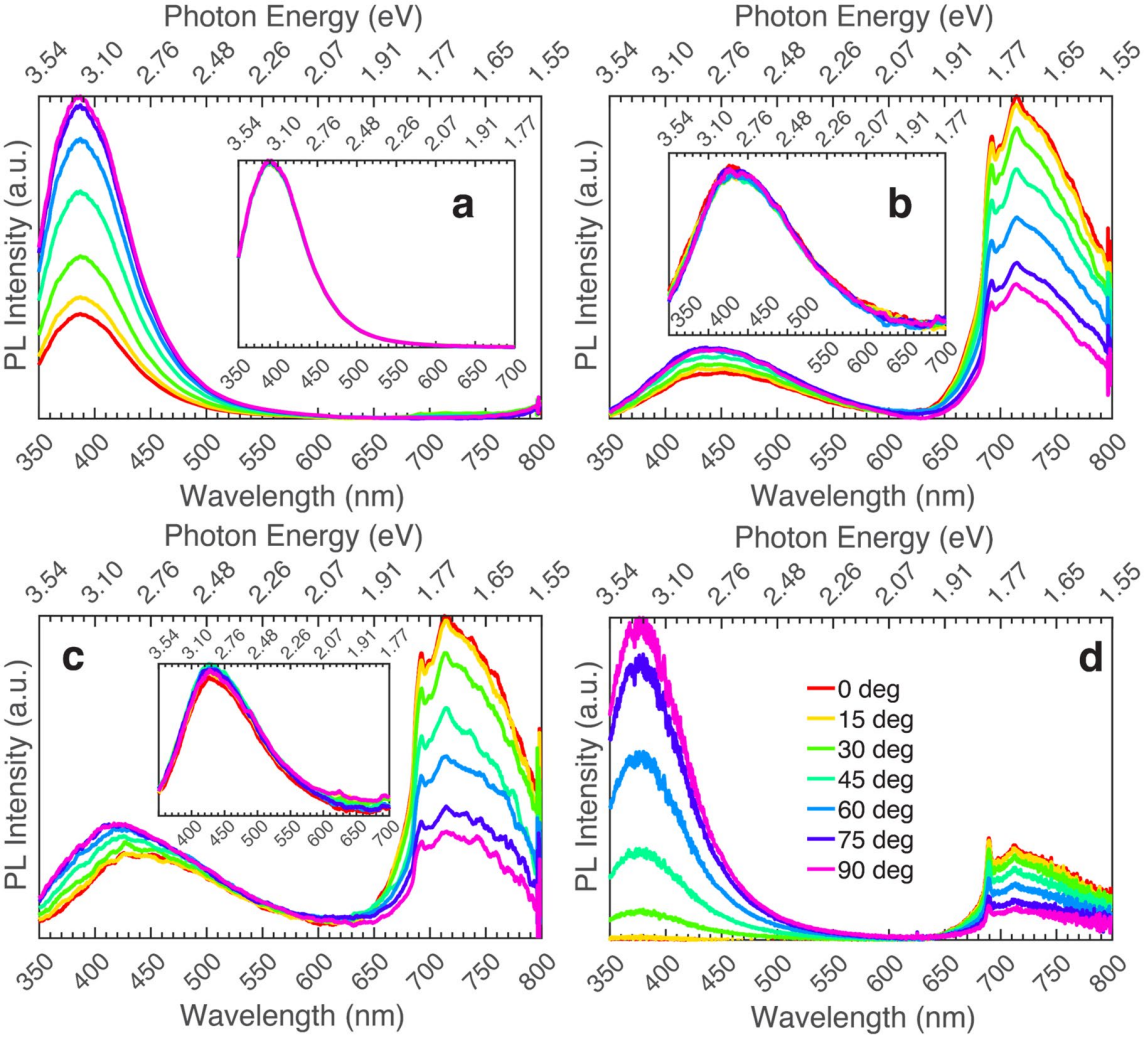
Polarized photoluminescence of different concentrations of AGO grown on (010) Fe-doped β-Ga_2_O_3_ excited at 254 nm (4.88 eV) except for (**d**). Insets show data for the same samples but excited at 235 nm (5.27 eV). (**a**) UID β-Ga_2_O_3_ grown on (010) Fe-doped β-Ga_2_O_3_, (**b**) 10% AGO grown on (010) Fe-doped β-Ga_2_O_3_, (**c**) 25% AGO grown on (010) Fe-doped β-Ga_2_O_3_, (**d**) 10% (100) bulk AGO excited at 235 nm (5.27 eV).

Shown in Fig. 5 is the polarized PL of four (010) oriented (Al_x_Ga_1−x_)_2_O_3_, of varying Al concentration grown on Fe-doped β-Ga_2_O_3_ samples, excited above and below the bandgap and is further compared to a (100) oriented bulk 10% AGO sample. The AGO films show several differences compared to the UID β-Ga_2_O_3_ film and bulk 10% AGO crystal. The UID β-Ga_2_O_3_ film and bulk AGO crystal have a stronger polarization dependence than the two AGO film samples. The AGO films have a smaller polarization difference between the maximum and minimum polarization states.

Furthermore, there is a shift in the dominant peak as the polarization changes for the 25% AGO sample. This polarization shift could be due to aluminum atoms preferring the tetrahedral sites within the monoclinic structure of β-Ga_2_O_3_ due to the atoms smaller size^46^. This could result in more types of one defect seen at the 0-degree polarization while a second defect rules the 90-degree orientation. But this is not seen in the bulk AGO crystal or 10% AGO film, which would be expected to show a similar polarization dependence shift in PL if this were the case. Thus, more studies are required to determine the cause of this shift.

**Figure 6:**
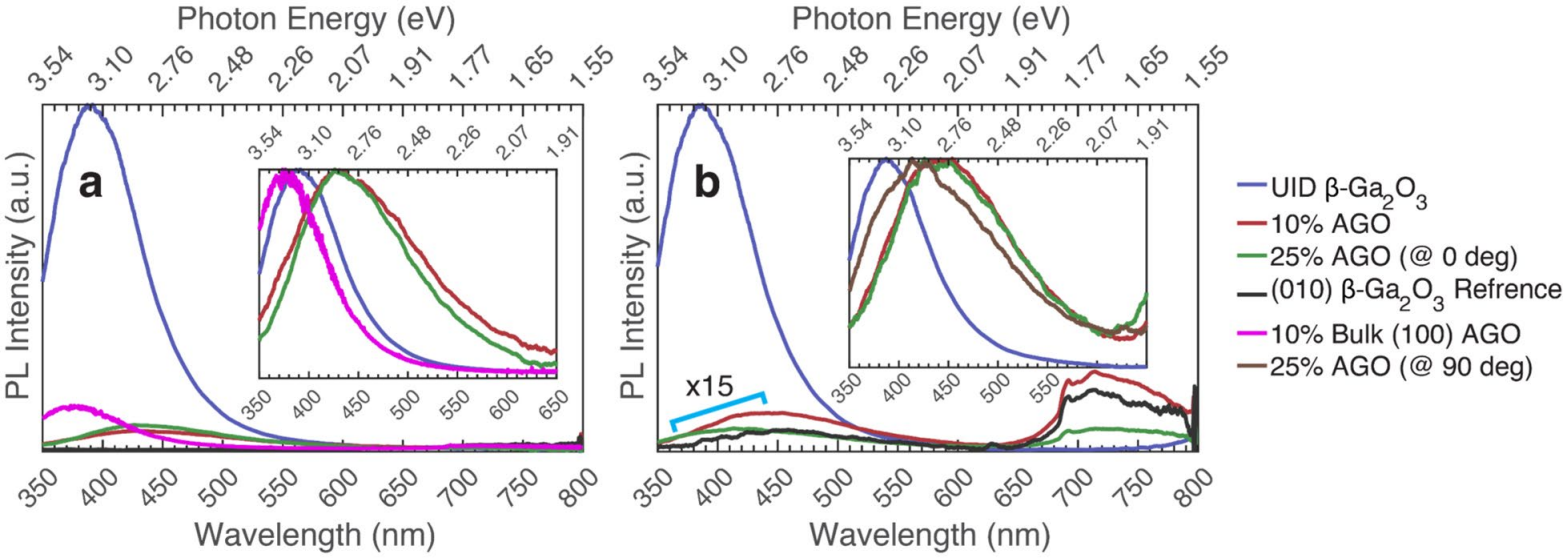
Photoluminescence comparison (taken at max polarization unless stated) of different concentrations of AGO grown on (010) Fe-doped β-Ga_2_O_3_ as well as compared to a 10% bulk (100) AGO sample. Inset depicts the same plot normalized. (**a**) Excited at 235 nm (5.27 eV), (**b**) Excited at 254 nm (4.88 eV).

Seen in Fig. 6 is a comparison of PL for AGO films with different Al concentrations grown on (010) Fe-doped β-Ga_2_O_3_, excited above and below the bandgap. There is a clear difference in intensity between UID β-Ga_2_O_3_ and all AGO samples below (267 nm or 4.64 eV) and above (235 nm or 5.27 eV) bandgap. This difference in intensity could be due to the aluminum generating more non-radiative recombination paths, quenching of radiative paths, or due to an increase in the radiative recombination lifetime. Furthermore, the intensity is greater for 25% AGO than 10% AGO when excited at 235 (5.27 eV) nm, as seen in Fig. 6a. This is due to the shift in bandgap that occurs in alloyed materials. Using Vegard’s law with a bowing parameter of 1.3^47–49^; β-Ga_2_O_3_ has a bandgap around 258.3 nm (4.8 eV), 10% AGO has a bandgap around 248 nm (5.0 eV), and 25% AGO has a bandgap around 234 nm (5.3 eV). On the other hand, the excitation used for Fig. 6a is 235 nm (5.27 eV). The maximum PL intensity for a sample is expected to be seen around its bandgap. At higher excitation energies, a strong absorption coefficient inhibits light from penetrating deep into the sample, so a higher percentage of photocarriers diffuse and recombine, causing lower radiative recombination. At lower excitation energies below the bandgap, the effects of bulk recombination and small optical thickness are accentuated, so there is less radiative recombination^36^. Since the excitation of 235 nm (5.27 eV) is closer to the bandgap of 25% AGO, there is a higher PL intensity for 25% AGO than for 10% AGO at that excitation. For the same reason, at an excitation of 254 nm (4.88 eV), as shown in Fig. 6b, a larger PL intensity for 10% AGO than 25% AGO is observed.

There is also a clear shift between the dominant emission peak between samples. The UID β-Ga_2_O_3_ film shows a dominant UV emission at 390 nm (3.18 eV), as for the Si-doped series and the (-201) bulk β-Ga_2_O_3_ crystal. The bulk AGO crystal also has a dominant UV emission, but around 380 nm (3.26 eV). On the other hand, the epitaxial AGO films have a dominant blue emission around 430 nm (2.88 eV) and are consistent with the (-201) oriented heteroepitaxial film grown on sapphire. This can again be explained by the extended defects found throughout the sample.

**Figure 7:**
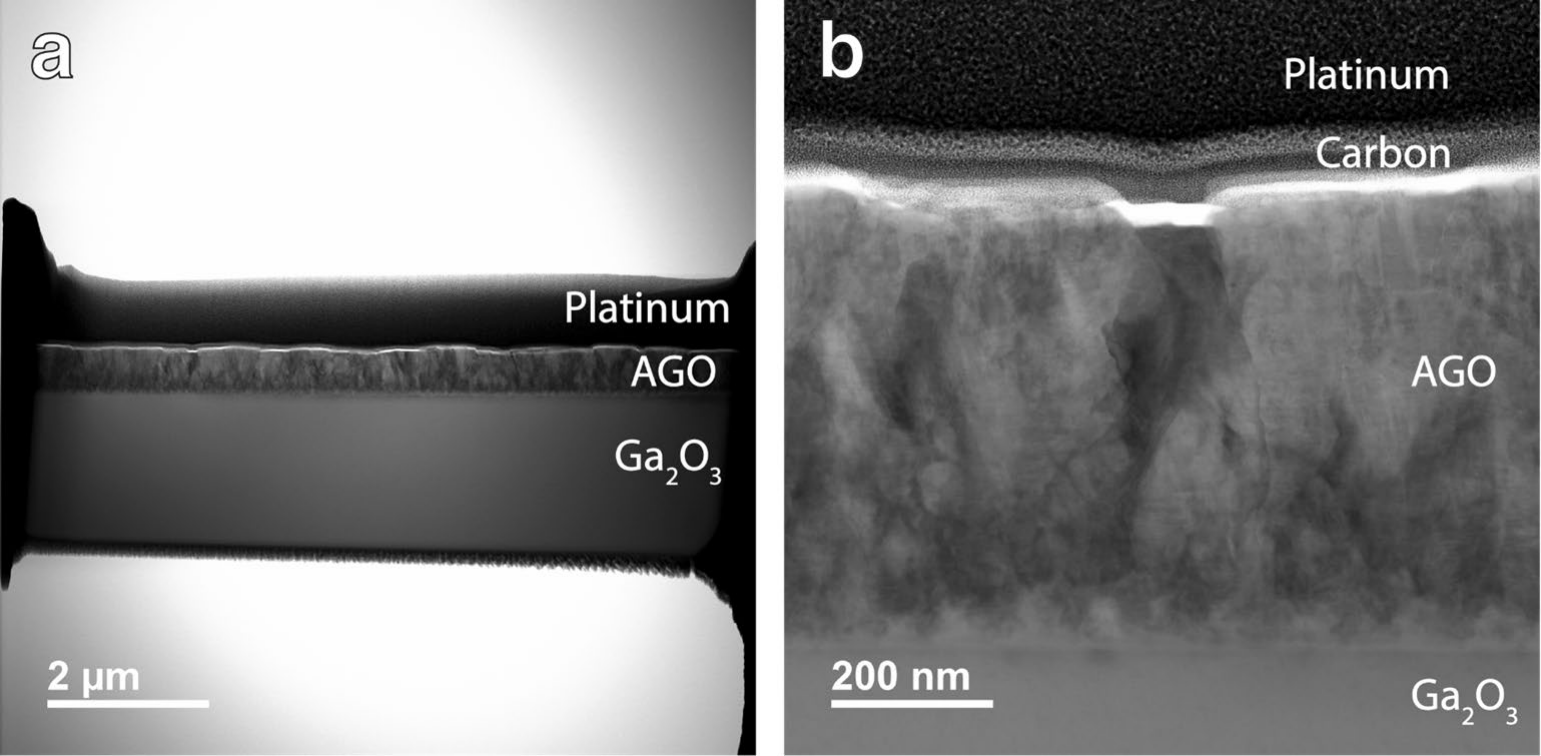
Darkfield STEM of 25% AGO grown on (010) Fe-doped β-Ga_2_O_3_. (**a**) Overview of the sample, (**b**) Section zoomed in.

TEM was done on the 25% AGO film grown on (010) oriented Fe-doped β-Ga_2_O_3_ using the JEOL JEM 2800 Scanning Transmission Electron Microscope. The dark field image is shown in Fig. 7. Even though the films were grown on (010) oriented β-Ga_2_O_3_ and tend to be homogeneous, the AGO films analyzed here, which were produced on the same substrate, show many extended defects as seen in the Fig. 7 Scanning TEM (STEM) image. This is likely caused by the high growth rate used to make the films thicker, which caused the epitaxy to break down. Furthermore, there is no difference between the 10% AGO and 25% AGO dominant blue PL emission, even though the number of point defects has changed between samples (and some of the growth conditions to accommodate for the desired aluminum concentration). There is a decrease in broadening from 10% AGO to 25% AGO, but this broad PL resembles that of the other defective samples. Again, the only real difference between UV dominant and blue dominant samples is that the blue dominant samples are riddled with extended defects, further revealing a correlation between a blue-centered PL spectrum and a large density of extended defects.

**Figure 8:**
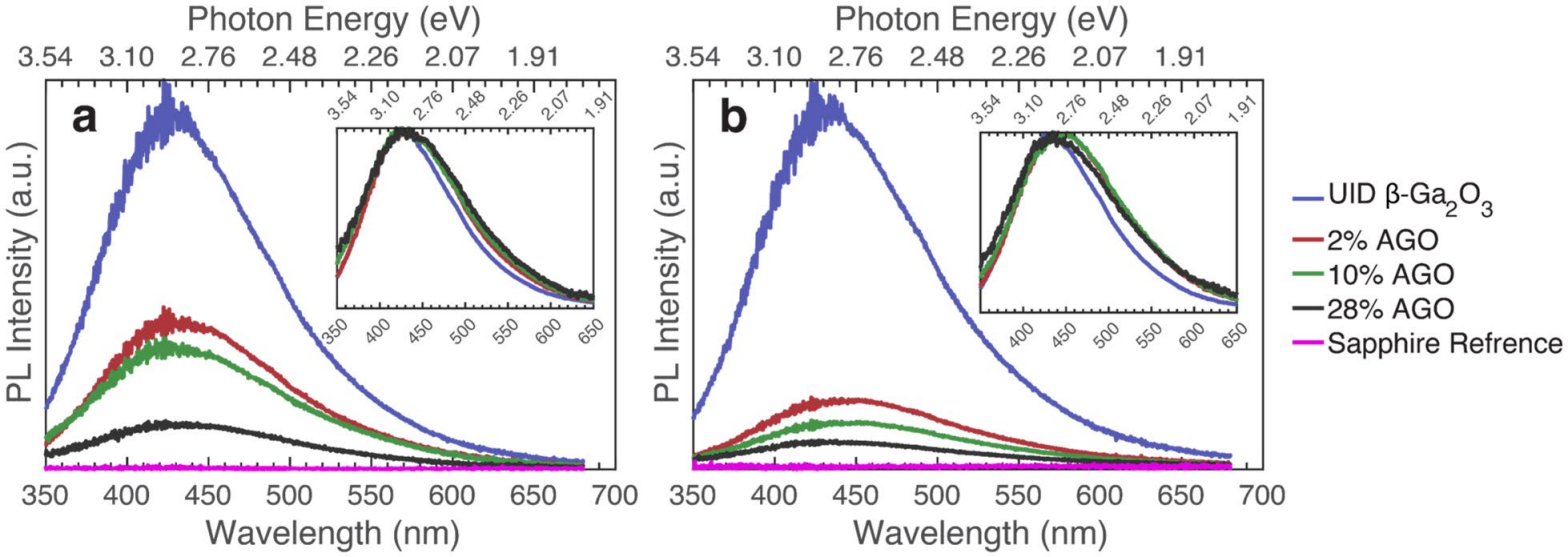
Photoluminescence comparison of different concentrations of AGO grown on c-plane sapphire. Inset depicts the same data smoothed using a 5-point moving average and then normalized to the peak value. (**a**) Excited at 236 nm (5.25 eV), (**b**) Excited at 254 nm (4.88 eV).

Lastly, the four samples in the AGO series grown on sapphire show consistent PL results with what has previously been seen. This is shown in Fig. 8, where the PL for AGO samples grown on sapphire with different Al concentrations are shown (excitation above and below bandgap). The series contains (-201) oriented 0%, 2%, 10%, and 28% AGO films grown on sapphire. No polarization dependences at any excitation were observed due to rotational domains in the (-201) oriented β-Ga_2_O_3_ film grown on sapphire. The dominant emission is in the blue, with the emission peak occurring at around 430 nm (2.88 eV). This is the same for all samples in this series. This is also consistent with the position of the dominant peak seen for the AGO films grown on (010) β-Ga_2_O_3_ and the (-201) oriented heteroepitaxial film grown on sapphire. Again, this is likely due to extended defects seen throughout the film, developed from a combination of the poor-quality film an alloy generates as well as a poor-quality film that is caused by growing on sapphire. This correlates with TEM images showing poor AGO films grown on sapphire discussed in previous papers^42,50–53^. Other observations seen in Fig. 8, which compares all the AGO films grown on sapphire, include the intensity decreasing with increasing aluminum concentration. This could be due to an increase in the electron–phonon coupling, as discussed for the other AGO samples. This could also explain why UID β-Ga_2_O_3_ PL does not extend so far into the blue and green emission as the AGO samples do. Another explanation could be due to the appearance of aluminum atoms creating new recombination paths that generate more blue and green emissions, though more study is necessary to determine the specific processes.

## Conclusions

**Figure 9:**
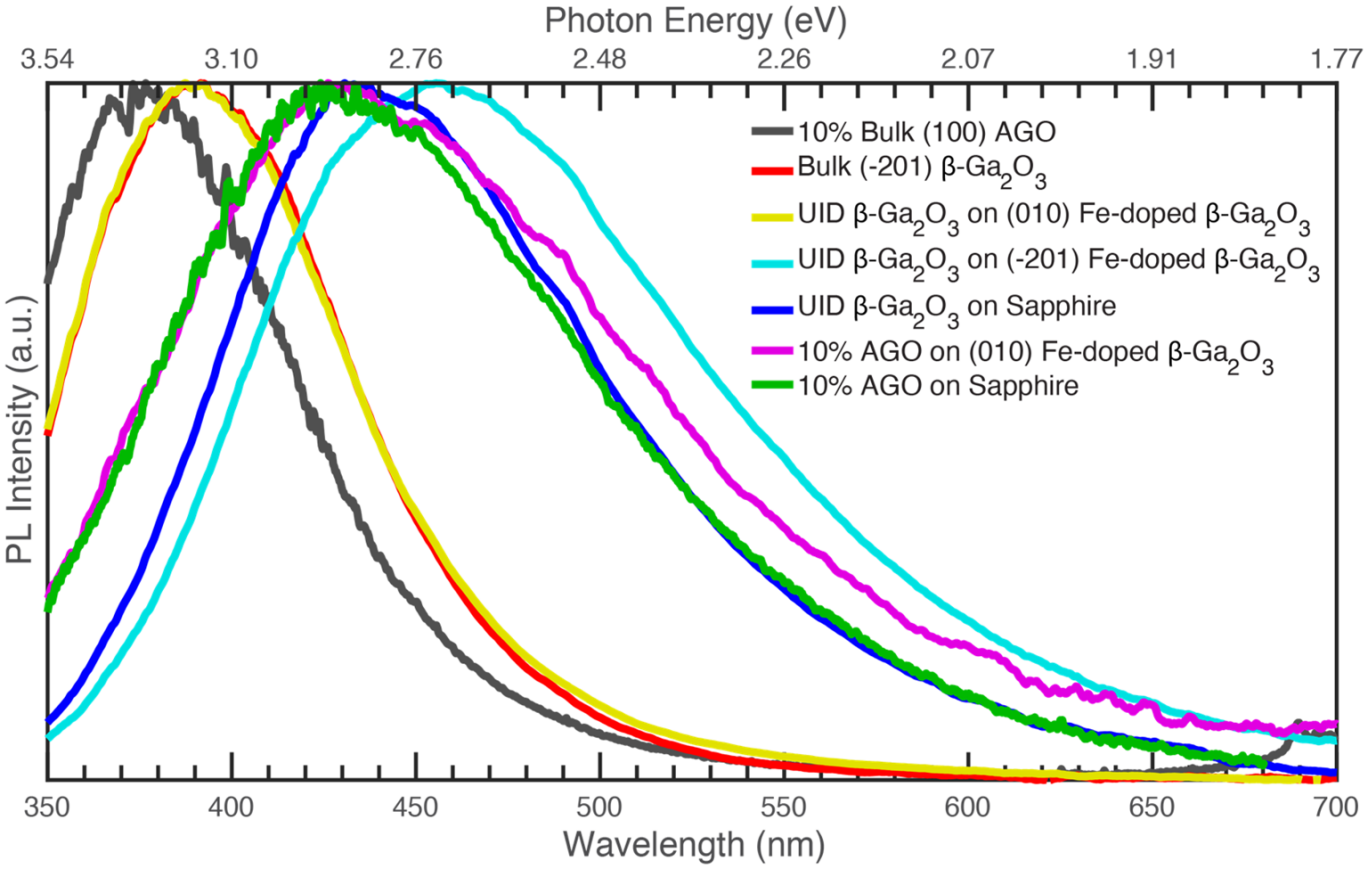
Normalized photoluminescence (taken at max polarization) comparison of all samples previously discussed excited at 235 nm (5.27 eV). The AGO grown on the sapphire sample was excited at 236 nm (5.25 eV), and the data were smoothed using a 5-point moving average before being normalized to the peak intensity.

Si-doped β-Ga_2_O_3_ grown on (010) β-Ga_2_O_3_ yields homogeneous crystalline films with a low density of extended defects and a dominant UV emission in PL. This is the same as what is seen for bulk crystals, including (-201) β-Ga_2_O_3_. Heteroepitaxial and homoepitaxial (-201) β-Ga_2_O_3_ films show consistent PL features with dominant blue emission and small UV luminescence. The films’ poor quality is likely the reason for this shift in the dominant emission, as observed when comparing UID films grown on (010) and (-201) bulk β-Ga_2_O_3_ crystals with comparable growth conditions. AGO for both homoepitaxial and heteroepitaxial samples also shows consistent blue centered PL with no UV emission observed. All these samples show extended defects throughout a poor-quality film. As such, we have hypothesized that extended defects have an essential role in the PL emission of β-Ga_2_O_3_ and AGO films. Homogeneous films with no extended defects or stacking faults and bulk crystals will yield a UV dominant PL emission. Samples of poor film quality that have stacking faults and extended defects will have a dominant blue PL emission. This is clearly shown in Fig. 9, which compares representative samples chosen from all the series previously discussed (excited at 235 nm or 5.27 eV). As seen, bulk crystals (both β-Ga_2_O_3_ and β-Al_0.2_Ga_1.8_O_3_, i.e., 10% AGO) and β-Ga_2_O_3_ films grown on (010) β-Ga_2_O_3_ all have a similar dominant UV peak PL signature likely because all samples are of good quality with minimal extended defects.

On the other hand, β-Ga_2_O_3_ films grown on sapphire, AGO films grown on (010) β-Ga_2_O_3_, and AGO films grown on sapphire all show the same dominant blue emission, even with differing defects and growth conditions. The only fundamental similarity for those samples is that all have proven to have poor quality films with large densities of extended defects, which do not necessarily have the same associated defect states as isolated point defects. β-Ga_2_O_3_ films grown on sapphire have extended defects developing from at least rotational domains. And films grown on (-201) β-Ga_2_O_3_ show an even more significant shift for the dominant blue emission. Again, the major difference between β-Ga_2_O_3_ films grown on (-201) β-Ga_2_O_3_ and β-Ga_2_O_3_ films grown on (010) β-Ga_2_O_3_ is that the (-201) β-Ga_2_O_3_ film exhibits a large density of extended defects which could arise from a chain of point defects or dislocations, stacking faults, interfaces, etc. And, it can be difficult to pinpoint the exact mechanisms as any defect considered can result in a very wide luminescence band, even near 0 K. That said, follow-up studies are needed to better understand the specific processes involved in these samples to understand the broadening and shifts seen in Fig. 9 and conclusively determine what recombination processes are dominant within the different series of samples.

## Supplementary Information

Supplementary Information.
