# Supplementary material for: Effect of extended defects on photoluminescence of gallium oxide and aluminum gallium oxide epitaxial films
Source: Sci Rep. 2022 Feb 25;12:3243. doi: 10.1038/s41598-022-07242-z (PMC8881628; doi:10.1038/s41598-022-07242-z)
Supplement: Supplementary file 1 — Supplementary Information. [file 41598_2022_7242_MOESM1_ESM.pdf]

Effect of extended defects on photoluminescence of gallium oxide and aluminum gallium oxide epitaxial films

*Jacqueline Cooke[1], Praneeth Ranga[1], Jani Jesenovec[4, 5], John S. McCloy[4, 5], Sriram Krishnamoorthy[3], Michael A. Scarpulla[1, 2], and Berardi Sensale-Rodriguez[1]**

[1]Department of Electrical and Computer Engineering, The University of Utah, Salt Lake City, Utah 84112, USA
E-mail: berardi.sensale@utah.edu

[2]Department of Materials Science and Engineering, The University of Utah, Salt Lake City, Utah 84112, USA

[3]Materials Department, University of California, Santa Barbara, Santa Barbara, California 93106-5050, USA

[4]Institute of Materials Research, Washington State University, Pullman, Washington 99164-2920, USA

[5]Materials Science & Engineering Program, Washington State University, Pullman, Washington, 99164, USA

Supplementary Information

Literature review on β-Ga_2O_3 photoluminescence:

The discussion on identifying the processes of PL emission for β-Ga_2O_3 has been ongoing since 1969 when Herbert *et al.* first measured PL from a single crystal, proposing that vacancies or impurity ions in the crystal served as energy traps and emission centers [5]. Since then, there have been many studies on the causes of observed UV, blue, and green emission peaks. This debate re-emerged around 2000 when crystal growth breakthroughs led to high-quality single crystals and thus superior samples [6]. In 2008, Shimamura *et al.* proposed that the quenching of the UV peak, observed in previous reports, was caused by samples that were not pure enough and that the UV peak was caused by a self-trapped exciton (STEX). The blue peak was attributed to the recombination of donors and acceptors at intrinsic vacancy centers. Furthermore, from the Gaussian model developed from the PL data, a UV' peak was discerned for the first time and attributed to the same processes as the UV peak [7]. In 2011, Wakai *et al.* observed that the PL of β-Ga_2O_3 was suppressed by Cr-doping, which was hypothesized to act as a non-radiative center

for photoexcited carriers. It was argued that the UV peak was due to band-to-band excitations, while the blue peak was due to energy-dependent relaxations of donor-acceptor pairs due to a polarization-dependent maximum at the fundamental absorption edge [8]. In the same year, Yamaga *et al.* attributed the UV emission to band-to-band excitation due to intrinsic rather than unintended impurity centers, since localized centers interact strongly with phonons and a similar observation of self-trapping holes in SiO_4 and AlO_4 tetrahedra was determined through electron-spin resonance (ESR). A self-trapped electron (STE) and bound electron was one explanation for this, or self-trapped hole (STH) in GaO_4 through strong electron-phonon interaction was another. The green peak was attributed to the recombination of electrons in a cluster of oxygen vacancies and trapped holes with electrons flowing freely in clusters and hopping between clusters using thermal energy [9].

Later work by Onuma *et al.* in 2013 determined that the blue peak intensity strongly depended on resistivity and was connected to oxygen vacancies and doped impurities for donor-acceptor pairs. The UV band was attributed to the recombination of free electrons and STH, since it was found to be impurity independent [10]. In 2015, Yamaoka *et al.* studied the Stokes shift and Urbach tail to determine the stability of STEX and found that exciton-phonon coupling occurs. The broad UV PL band was attributed to STEX emission for this reason [11]. In 2017, Bordun *et al.* observed four emission peaks; deep UV, UV, and two blue peaks. The blue peaks were attributed to the interaction of oxygen and gallium vacancies with broadening owing to the distance between the donor and acceptor with strong electron-phonon coupling. The UV peak was attributed to the recombination of bound excitons in quantum wells formed by acceptor clusters localized in the $(GaO_6)^{9-}$ structure and relaxed into a lower vibrational state. A linear electro-phonon coupling model was used to verify that the deep UV peak was formed by deep quantum wells of acceptor clusters [12]. Oda *et al.* also reported four emission peaks; deep UV, UV, blue, and green. The green peak was determined to have a weak absorption that comes from a slower decay component (using time-resolved PL) of electronic transitions from valence bands to impurity levels lying below the conduction band [13]. In 2018, Ho *et al.* created a hybrid model for point defects in β-Ga_2O_3 describing the UV peak as STH states due to previously seen small hole polarons, which make up two UV peaks. The blue peak was assigned to singly negative Ga-O divacancies (V_{Ga}-V_O), and the green peak to interstitial oxygen atoms, which were supported by observed polarization properties and intensity variations on carrier concentration and stoichiometry [14]. Huynh *et al.* determined that the UV peak was quenched at high temperatures and consists of weakly bound electrons in STEX. The blue peak was quenched due to thermal activation of non-radiative recombination centers and was argued to be related to an oxygen vacancy donor defect coupled to the crystal lattice. This was due to the determined activation energy, which was much smaller than the activation energy of oxygen vacancy defects [15]. Liu *et al.* improved on the discussion describing that the blue band could be deconvoluted into two peaks. The green peak was related to the recombination of bound exciton with the hole trapped at one of three different defects; gallium-oxygen divacancies, gallium vacancies (octahedral site), and oxygen interstitials, respectively [16]. In the same year, Wang *et al.* showed that the UV peak could also be

deconvoluted into two UV peaks of which the emission of each originates from transitions between the conduction band electrons and two differently-populated STH states from the O_I and O_{II} polaron sites of the β-Ga_2O_3 lattice. This was seen as a shift in the UV peak depending on polarization at any excitation [17].

More recently, in 2019, Berencén *et al.* confirmed that the UV peak was due to STH in a small polaron state between two oxygen sites, as was described by Wang *et al*. Furthermore, they verified the theories of other reports that described the two blue peaks and green peak originating from gallium-oxygen vacancy pairs (1- charge state), gallium vacancies (2- charge state), and neutral oxygen interstitials (originating by splitting with O_I), respectively. Furthermore, red emission was attributed to neutral oxygen vacancies [18]. Cui *et al.* determined that electrons could be captured by Ga_I (Ga^{3+}), instead of a decrease in oxygen vacancies, causing the blue peak to decrease when annealed. This was determined by observing intensity changes as seen in Raman spectroscopy [19]. In the same year, Hany *et al.* disagreed with the notion that the UV peak comes from STH, instead stating that the UV and blue peaks both come from defect to defect transitions due to the peaks being temperature insensitive. Four UV peaks were argued to arise from electrons of the donor band, including oxygen vacancy, gallium interstitial, or dopant (rather than conduction band) falling to gallium and oxygen vacancy pair acceptor or STH. Furthermore, the blue and main UV peaks were ascribed as a donor-acceptor pair with an oxygen vacancy as the donor and gallium vacancy or Fe-dopant as the acceptor, respectively. This was due to the significant decrease of these peaks when annealed in air [20]. In 2020, Huso *et al.* used PL mapping and discovered that emission centers were not distributed homogeneously throughout a β-Ga_2O_3 single crystal but were seen in specific, localized regions that could not be identified [21]. In 2021, Cho *et al.* further revealed that gallium and oxygen vacancies in β-Ga_2O_3were non-uniformly distributed from the sample surface to the inside of the sample [22]. This was further verified by Jesenovec *et al.,* whose positron results agreed that there was a stronger compensation of the donors near the surface for gallium vacancies formed in β-Ga_2O_3 annealed in oxygen [23]. The anisotropic valence band leads to PL being highly polarized. This was due to the symmetry of gallium and oxygen vacancy transitions towards the valence band. Furthermore, PL was argued to be extrinsic, involving gallium and oxygen vacancies for blue and UV emissions, respectively. The transitions were proposed to arise from radiative recombination of room-temperature-stable excitons bound to a vacancy, rather than STEX, due to absorption power dependence based on a polaron model [22].

In the most recent work, Zhang *et al.* decomposed the PL emission into ten separate peaks based on previous reports and due to the strong and different anisotropic behavior of all the PL bands; two UV peaks, five blue peaks, three green peaks, and a yellow peak. The first UV peak was attributed to a phonon-assisted transition from the conduction band to a STH. The second UV peak and first two blue bands originate from free electrons detachment by STH at the oxygen vacancy donor position; O_I, O_{II}, O_{III}, respectively. Polarization of the UV band was determined by the spatial distribution of STH located at oxygen sites, of which the density of O-sites was higher in specific orientations. The third blue band was from oxygen interstitial to STH. The fourth and

fifth blue peaks were thought to differ from a change in acceptors, from oxygen interstitials to an acceptor located at gallium sites Ga_I and Ga_{II}, respectively. Due to the different symmetry of the electron density distribution, a specific orientation occurred for recombination. The first green peak was thought to come from oxygen vacancy donor (O_{II}) to either Ga_{II} or Mg dopant. The third green peak was thought to come from oxygen interstitial (O_I) to either Ga_{II} or Mg dopant. Lastly, the second green peak and yellow peak could not be identified [24].

Table S1. Summary of PL emission definitions from previous publications

Year	Author	UV	Blue	Green
2008	Shimamura [7]	STEX	Recombination of donors/ acceptors at intrinsic vacancy centers	
2011	Wakai [8]	Band-to-band excitations	Energy-dependent relaxations of donor-acceptor pairs	
2011	Yamaga [9]	Intrinsic band-to-band excitation (STE or STH)		
2013	Onuma [10]	Recombination of free electrons and STH	Oxygen vacancies and doped impurities for donor-acceptor pairs	
2015	Yamaoka [11]	STEX		
2017	Bordun [12]	UV: Recombination of bound excitons in quantum wells formed by acceptor clusters localized in the $(GaO_6)^{9-}$ structure and relaxed into a lower vibrational state<br><br>Deep UV: deep quantum wells of acceptor clusters	Oxygen and gallium vacancies	
2017	Oda [13]			Slower decay component of electronic transitions from valence bands to impurity levels below conduction band
2018	Ho [14]	2 peaks from STH	Singly negative Ga-O divacancies	Interstitial oxygen atoms
2018	Huynh [15]	Weakly bound electrons in STEX	Oxygen vacancy donor defect coupled to the crystal lattice	
2018	Liu [16]			Recombination of bound exciton with hole trapped at one of three different defects
2018	Wang [17]	Conduction band electrons at two different-populated STH states		
2019	Berencén [18]	STH	Gallium-oxygen vacancy pairs or gallium vacancy	Oxygen interstitials
2019	Hany [20]	Defect to defect transitions	Defect to defect transitions	

2021	Cho [22]	Radiative recombination of exciton bound to oxygen vacancies	Radiative recombination of exciton bound to gallium vacancies	
2021	Zhang [24]	Phonon-assisted transition from the conduction band to STH<br><br>2nd peak: Free electrons detachment by STH at the oxygen vacancy donor position	First 2 peaks: Free electrons detachment by STH at the oxygen vacancy donor position<br><br>3rd: Oxygen interstitial to STH<br><br>4th and 5th: Change in acceptors, from oxygen interstitials to an acceptor located at gallium sites Ga_I and Ga_{II}, respectively	Oxygen vacancy donor (O_{II}) to either Ga_{II} or Mg dopant<br><br>3rd: Oxygen interstitial (O_I) to either Ga_{II} or Mg dopant

References (number consistent with reference number in main manuscript text):

[5] Herbert, W. C., Minnier, H. B. & Brown Jr., J.J. Self-Activated Luminescence of β-Ga_2O_3. *J. Electrochem. Soc.: Solid State Science.* **116**, 1019-1021 (1969).

[6] Tomm, Y., Reiche, P., Klimm, D., Fukuda, T. Czochralski grown Ga_2O_3 crystals. *J. Cryst. Growth.* **220**, 510-514 (2000).

[7] Shimamura, K., Víllora, E. G., Ujiie, T. & Aoki, K. Excitation and photoluminescence of pure and Si-doped β-Ga_2O_3 single crystals. *Appl. Phys. Lett.* **92**, 201914 (2008).

[8] Wakai, H., Sinya, Y. & Yamanaka, A. Effect of Cr^{3+} ions on Optical Properties in β-Ga_2O_3 Semiconductor. *Phys. Status Solidi C.* **8**, 537-539 (2011).

[9] Yamaga, M., *et al.* Polarization of optical spectra in transparent conductive oxide β-Ga_2O_3. *Phys. Status Solidi C.* **8**, 2621-2624 (2011).

[10] Onuma, T., *et al.* Correlation between blue luminescence intensity and resistivity in β-Ga_2O_3 single crystals. *Appl. Phys. Lett.* **103**, 041910 (2013).

[11] Yamaoka, S. & Nakayama, M. Evidence for formation of self-trapped excitons in a β-Ga_2O_3 single crystal. *Phys. Status Solidi C.* **13**, 93-96 (2016).

[12] Bordun, O. M., Bordun, B. O., Kukharskyy, I. Y. & Medvid, I. I. Photoluminescence properties of β-Ga_2O_3 thin films produced by ion-plasma sputtering. *J. Appl. Spectrosc.* **84**, 46-51 (2017).

[13] Oda, H., Kimura, N., Yasukawa, D., Wakai, H. & Yamanaka, A. Time-resolved spectroscopy of luminescence in a wide gap Si-doped β-Ga_2O_3. *Phys. Status Solidi. A.* **214**, 1600670 (2017).

[14] Ho, Q. D., Frauenheim, T. & Deák, P. Origin of photoluminescence in β-Ga_2O_3. *Appl. Phys. Rev. B.* **97**, 115163 (2018).

[15] Huynh, T. T., Lem, L. L. C., Kuramata, A., Phillips, M. R. & Ton-That, C. Kinetics of charge carrier recombination in β-Ga_2O_3 crystals. *Phys. Rev. Mater.* **2**, 105203 (2018).

[16] Liu, C., *et al.* Irradiation effects on the structural and optical properties of single crystal β-Ga_2O_3. *Semicond. Sci. Technol.* **33**, 095022 (2018).

[17] Wang, Y., *et al.* Incident wavelength and polarization dependence of spectral shifts in β-Ga_2O_3 UV photoluminescence. *Sci. Rep.* **8**, 18075 (2018).

[18] Berencén, Y., *et al.* Structural and optical properties of pulsed-laser deposited crystalline β-Ga_2O_3 thin films on silicon. *Semicond. Sci. Technol.* **34**, 035001 (2019).

[19] Cui, H., *et al.* Analysis on the electronic trap of β-Ga_2O_3 single crystal. *J. Mater. Sci.* **54**, 12643-12649 (2019).

[20] Hany, I., *et al.* Low temperature cathodoluminescence study of Fe-doped β-Ga_2O_3. *Mater. Lett.* **257**, 126744 (2019).

[21] Huso, J., McCluskey, M. D., Yu, Y., Islam, Md M. & Selim, F. Localized UV emitters on the surface of β-Ga_2O_3. *Sci. Rep.* **10**, 21022 (2020).

[22] Cho, J. B., *et al.* Highly asymmetric optical properties of β-Ga_2O_3 as probed by linear and nonlinear optical excitation spectroscopy. *J. Phys. Chem. C.* **125**, 1432-1440 (2021).

[23] Jesenovec, J., *et al.* Gallium vacancy formation in oxygen annealed β-Ga_2O_3. *J. of Appl. Phys.* **129**, 245701 (2021).

[24] Zhang, N., *et al.* Anisotropic luminescence and third-order electric susceptibility of Mg-doped gallium oxide under the half-bandgap edge. *Opt. Express.* **29**, 18587-18600 (2021).
